# Analytical Pyrolysis of Sunflower Seed Husks: Influence of Hydrogen or Helium Atmosphere and Ex Situ Zeolite (HZSM-5) Catalysis on Vapor Generation

**DOI:** 10.1021/acsomega.4c09416

**Published:** 2025-01-24

**Authors:** Arthur T. F. A. Araújo, Anderson L. de Menezes, Cássia R. Cardoso, Daniel A. Cerqueira

**Affiliations:** † Department of Chemistry, Universidade Federal do Triângulo Mineiro, Uberaba, Minas Gerais 38064-200, Brazil; ‡ Faculty of Chemical Engineering, Universidade Federal de Uberlândia, Uberlândia, Minas Gerais 38400-902, Brazil; § Department of Food Engineering, Universidade Federal do Triângulo Mineiro, Uberaba, Minas Gerais 38064-200, Brazil

## Abstract

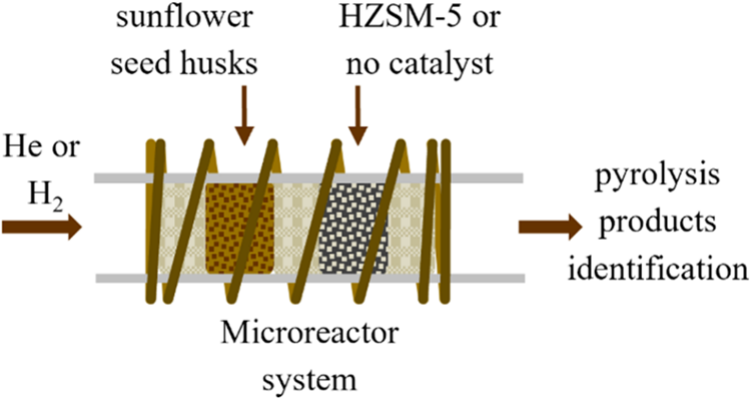

Several techniques are available for converting biomass into energy and/or valuable chemicals. This study aimed to perform the pyrolysis of sunflower seed husks (SSH) to analyze volatile products. Analytical fast pyrolysis experiments of SSH were carried out under four different conditions: pure SSH in a helium atmosphere (CGHe); pure SSH in a hydrogen atmosphere (CGHi); SSH with ex situ HZSM-5 (proton exchanged Zeolite Socony Mobil-5) zeolite in a helium atmosphere (CG-ZeHe); and SSH with ex situ HZSM-5 zeolite in a hydrogen atmosphere (CG-ZeHi). For each experimental condition, experiments were carried out at 450, 550, and 650 °C, and the volatile products were analyzed in a Py-GC/MS (micropyrolysis coupled to gas chromatography–mass spectrometry) system. The results showed that the zeolitic catalyst presented great effect on the organic functions in the volatiles and the selectivity distribution, especially for the formation of hydrocarbons. This catalyst could promote deoxygenation reactions of volatiles, enabling the generation of improved vapors. In CG-ZeHi, the production of hydrocarbons achieved a maximum of 42% peak area at 550 °C.

## 1 Introduction

The global population has been rapidly increasing in recent decades, leading to a higher demand for energy and products. Currently, the main energy sources in the world are fossil fuels, such as coal, oil, and natural gas. Oil is one of the main sources of organic chemicals in modern society. However, the increase in population and demand for energy and products is contradictory since the reserves of fossil fuels are finite. In addition, they are not environmentally friendly since the consumption of fossil fuels can harm the environment, leading to global warming, pollution, and damage to the ecological balance.^1−3^

An alternative to mitigate the environmental problems and allow sustainable development is to use biomass as a source of energy and value-added organic chemicals. Wood and other forms of biomass are some of the main renewable energy resources available and provide a renewable source of solid, liquid, and gaseous fuels.^4,5^ Several methods are available to convert biomass into energy. Thermal conversion is one of the main methods to recover energy from biomass by using combustion, gasification, and pyrolysis. The selection of one of these processes will depend on the type and quantity of biomass feedstock available, the objective of thermal conversion, environmental standards, economic conditions, and other factors.^6^

Pyrolysis is one of the ways to convert biomass into energy. This process occurs by direct thermal degradation of biomass in the partial or total absence of oxygen to produce gases, solids, and liquids. There are several types of pyrolysis processes. They depend on the reactor used, the gaseous atmosphere, and the residence time inside the reactor. Pyrolysis is a flexible process concerning raw materials and product delivery. Biomass pyrolysis generates water, biochar (carbonaceous solid), oils or tars, and permanent gases, such as methane, hydrogen, carbon monoxide, and carbon dioxide. The nature of changes in pyrolysis depends on the material, the final temperature, and the heating rate.^7,8^

Fast pyrolysis is one of the most promising processes in which lignocellulosic biomass is converted mainly into liquid products. Fast pyrolysis is the thermal degradation of organic materials in the absence of oxygen, at heating rates of approximately 500 °C/s, and a final temperature of 500 to 600 °C. Rapid heating breaks down large biomass molecules into smaller ones, releasing volatile products. The volatile products are quickly cooled down to room temperature, generating a brownish liquid fuel called bio-oil or biopetroleum.^9^

The fast pyrolysis liquids are dark brown and fluid, and the viscosity is similar to a medium fuel oil. Bio-oil is also sensitive to high temperatures when it undergoes chemical changes, so its distillation is challenging. Its lower energy value is around 17 MJ/kg compared to 42–44 MJ/kg for conventional fuel oil. It also presents a high oxygen content, a high acidity, and chemical instability. Therefore, applying bio-oil in the industrial and transport sectors is challenging. Bio-oil can replace fuel oil or diesel in several applications, such as boilers, furnaces, engines, and turbines. There are also a variety of chemicals that can be extracted or derived from bio-oil, including food flavorings, resins, agrochemicals, fertilizers, and emission control agents.^9,10^

Some methods have been developed to improve bio-oil quality such as catalytic fast pyrolysis, which directly integrates catalytic upgrading and pyrolysis of lignocellulosic biomass in a reactor. Catalysts play an important role in the efficiency of biomass pyrolysis by targeting specific reactions and reducing processing temperature and time. They affect the chemical composition and distribution of pyrolysis products. They can also reduce the oxygen content to proportions of 5 to 10%, resulting in a liquid oil with much less active oxygen-containing species and greater stability. The most important catalyst groups used in catalytic fast pyrolysis are zeolites, metal oxides, inorganic salt additives, carbon-based catalysts, and a wide combination of them.^11−14^

Hydropyrolysis is a technique to obtain less oxygenated bio-oil. It uses hydrogen gas (H_2_) as a reagent rather than an inert atmosphere. In fast hydropyrolysis, hydrogen produces radicals that react with volatiles released by biomass. This helps to break bonds within individual components, such as cellulose, hemicelluloses, and lignin, and between these components. Some oxygen is removed as water, carbon dioxide, or carbon monoxide. Char formation is inhibited. Free radicals and some compounds responsible for bio-oil instability are not formed. All of these conditions help to produce hydrocarbons and other special chemicals. Furthermore, hydropyrolysis processes can be carried out through catalytic or noncatalytic systems. Recent studies have shown that catalytic hydropyrolysis is a promising technique for producing liquid hydrocarbon fuels from lignocellulosic biomass.^9,15,16^

There are some advantages to using lignocellulosic biomass from agricultural waste as raw material to produce bio-oil and obtain higher value-added products. From an economic point of view, if the use of these agro-industrial residues reduces disposal costs and enables the use of cheaper raw materials for thermal conversion processes, the smaller volume of disposal of these residues and the possibility of their partial replacement by fossil fuels will contribute to more sustainable development.^17^

Sunflower (*Helianthus annuus* L.) is cultivated throughout the world. It is an annual species of dicotyledons from Asteraceae, the largest family of angiosperms, and originates from North America. This crop is one of the most important oilseeds in the world along with palm oil, soybeans, and rapeseed, and plays an important role in the world economy. It is one of the most important oilseeds that produces edible vegetable oil. It is extremely versatile, and practically all its parts can be explored. Sunflower seed husks (SSH) are intensely produced as waste from its grains processing. These SSHs are a source of biomass. They can be used in animal feed and solid fuel and pressed into chipboards for furniture manufacturing. The SSH can also be used in thermal convention processes, such as pyrolysis, to offer higher value-added products.^18−20^

Currently, several studies have focused on micropyrolysis and hydropyrolysis of lignocellulosic biomass to obtain products that can replace, at least in part, products from fossil fuels. Wu et al.^21^ investigated the conversion of sugar cane into furfural and levoglucosan with high selectivity. The authors promoted prior torrefaction of the biomass and, subsequently, conducted fast pyrolysis in a microreactor. Usino, Ylitervo, and Richards^22^ performed fast copyrolysis of palm kernel shells and sawdust in a micropyrolyzer to investigate the interactions between an agricultural residue and two woody biomass materials and their impact on the distribution of the primary product. Su et al.^23^ analyzed how HZSM-5 catalysts modified with metal oxide improve the formation of desirable hydrocarbons and decrease the formation of coke during the catalytic pyrolysis process of biomass under a hydrogen atmosphere. Jindal et al.^24^ subjected cotton stalk waste to catalytic hydropyrolysis using a micropyrolysis system coupled to gas chromatography–mass spectrometry (Py-GC/MS system) to analyze the influence of temperature and different catalytic configurations on the distribution of the product, mainly aromatic and aliphatic hydrocarbons.

This study investigated the pyrolysis of SSH, a kind of biomass for which there are still few studies on chemical conversions, as a method of using this agro-industrial residue to produce added value products. Fast micropyrolysis was performed, and its volatile products were analyzed under various conditions, such as modification of the atmosphere (He or H_2_) and application of a zeolite ex situ catalyst. The volatile product yields are also calculated for each condition. The main innovation of this work is the presentation and discussion of the effects of the reactive hydrogen atmosphere under catalysis.

## 2 Experimental Section

### 2.1 Materials

#### 2.1.1 Biomass

The SSH samples were provided by Parecis S/A, located in Campo Novo do Parecis, Mato Grosso state, Brazil. This company’s main activity is the production of vegetable oils and bran for animal feed using sunflower, soybeans, and cottonseed.

#### 2.1.2 Catalyst

The ammonium ZSM-5 zeolite catalyst was used and purchased from Alfa Aesar. This catalyst when undergoing a calcination process at 500 °C, under similar conditions to those reported by Li et al.^25^ and Santana, Menezes, and Ataíde,^26^ is transformed into its protonic form known as HZSM-5 zeolites or HZSM-5. The HZSM-5 zeolite is widely used due to the presence of strong acidic sites that lead to high yields of bio-oil and hydrocarbons.^27^

Santana, Menezes, and Ataíde^26^ characterized the HZSM-5 and established mass/mass composition in the SiO_2_/Al_2_O_3_ ratio of 23:1, a surface area of 425 m^2^/g, a pore volume of 0.25 cm^3^/g, and a density of 2.11 g/cm^3^. The acidic properties of ZSM-5 were analyzed using temperature-programmed ammonia desorption (NH_3_-TPD). The NH_3_-TPD analyses were performed using a Micromeritics Autochem 2920 II analyzer equipped with a thermal conductivity detector. Samples (0.1 g) were pretreated under a helium flow of 30 mL/min at 150 °C for 30 min. After pretreatment, the samples were cooled down to 100 °C and exposed to an ammonia flow for 60 min for ammonia adsorption. Thermal desorption of ammonia was carried out at a temperature range of 100–650 °C at a temperature increasing rate of 10 °C/min. The total acidity of the HZSM-5 was 1.23 mmol NH_3_/g.^26^

### 2.2 Experimental Procedure

The grinding, immediate analysis, chemical composition analysis, and calorific value of the SSH samples were previously carried out by Tibola^28^ and Tibola et al.^29^ The material was ground in a knife mill and sieved to achieve adequate particle size for subsequent analyses. The immediate analysis of SSH particles passing 65 mesh determined the moisture, volatile content, fixed carbon content, and ash content of the SSH samples. This analysis was performed in triplicate and followed the ABNT NBR 8112 standard.^30^ The content of extractives, lignin and holocellulose (cellulose and hemicelluloses), was determined according to Setter et al.,^31^ using SSH particles passing 16 mesh. The higher calorific value, which expresses the amount of energy of the sample per unit of mass, was estimated using the empirical correlation proposed by Yin,^32^ based on the values obtained in the immediate analysis.^28^

### 2.3 Analytical Micropyrolysis

The SSH analytical micropyrolysis experiments were conducted in two major blocks: fast pyrolysis and hydropyrolysis. Fast pyrolysis addressed conventional fast pyrolysis with pure SSH and catalytic fast pyrolysis, both configurations in an inert atmosphere (He). The hydropyrolysis also addressed pure SSH and a catalyst but in a reactive hydrogen atmosphere. These experiments are described as follows based on Santana, Menezes, and Ataíde.^26^

#### 2.3.1 Fast Pyrolysis

The analyses considered samples passed through a 100 mesh sieve to reduce thermal resistance to heat and mass transfer within the sample. Fast micropyrolysis experiments were carried out for pure SSH and ex situ HZSM-5 zeolite in a mass ratio of 1:1.^33−35^ For each sample, analyses were performed in duplicate to ensure the reproducibility of the results.

**Figure 1:**
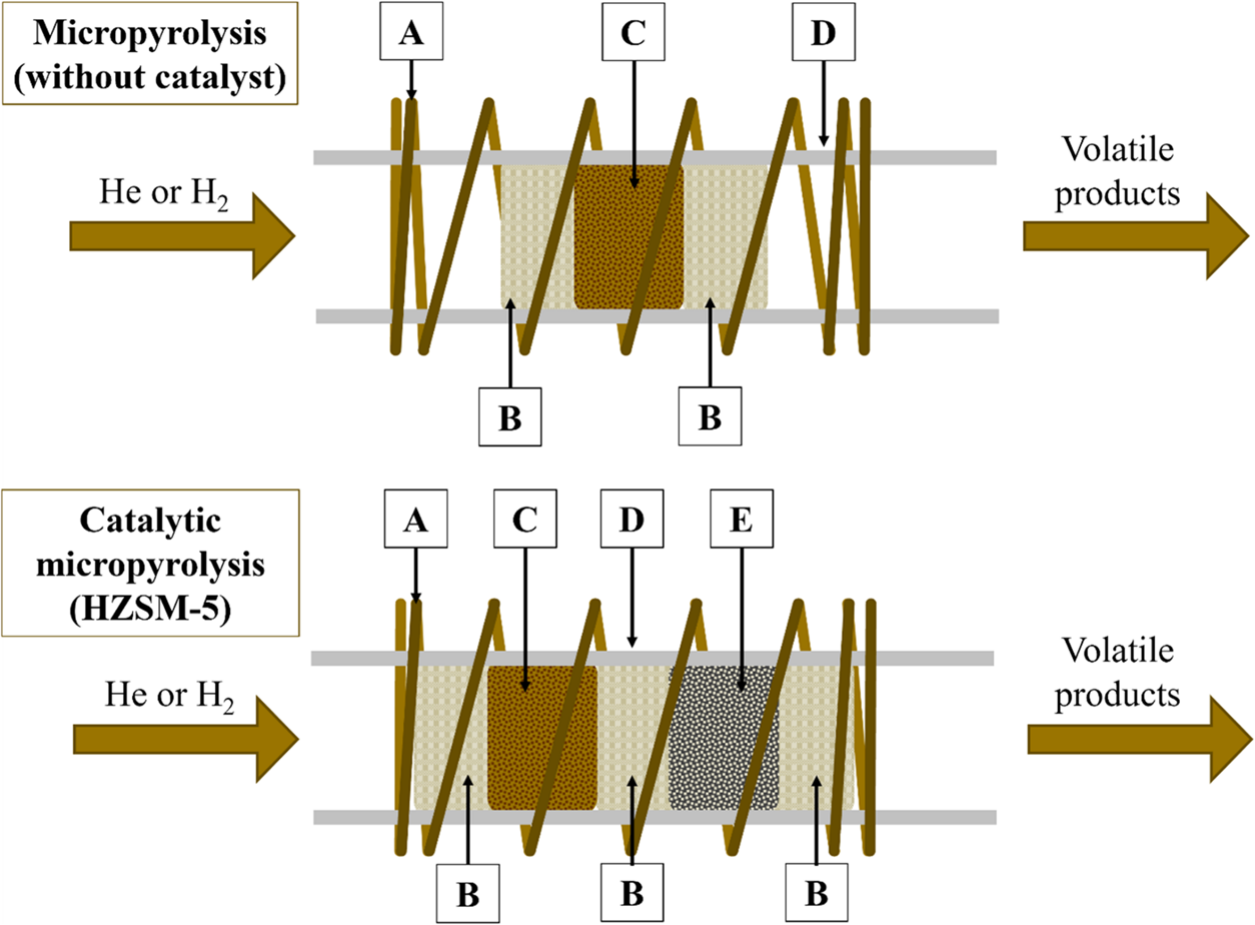
Schematic illustration of the CDS 5200 micropyrolyzer equipment. (A) Platinum resistance. (B) Quartz wool. (C) Sample (SSH). (D) Quartz capillary. (E) Catalyst (HZSM-5).

Micropyrolysis was carried out using CDS 5200 equipment. An amount of each sample was inserted into a quartz capillary, with a diameter of 2 mm, together with quartz wool (inert) on both sides. Quartz wool was used to ensure that the sample remained in the center of the capillary. Then, the assembly was positioned close to a platinum resistance and inserted into the micropyrolyzer. We considered the following sequence for ex-situ HZSM-5 zeolite catalysis samples: quartz wool, SSH, quartz wool, HZSM-5, quartz wool. Figure 1 illustrates the configurations of conventional (without catalyst) and catalytic micropyrolysis. The inert gas was helium 5.0 (purity 99.999). The heating rate was 20 °C/ms. The reaction temperatures were 450, 550, and 650 °C. After reaching the final temperature, the resistance remained heated for 10 s.

The micropyrolyzer interface was programmed to remain at 75 °C in stand-by condition and to be heated to 300 °C during pyrolysis. After heating, the temperature of 300 °C was maintained for 1 min. The pyrolyzer/GC transfer line and pyrolyzer valve remained at 280 °C.

The volatile products formed in the micropyrolysis of each sample were analyzed using gas chromatography–mass spectrometry (GC/MS QP2010 Plus). A Rtx-1701 capillary column (60 m × 0.25 mm × 0.25 μm) was used. Helium gas (99.999 purity) was used as carrier gas with a column flow of 1 mL/min. During the analyses, the injector temperature was maintained at 250 °C, the interface temperature at 270 °C, and the ionization source temperature at 275 °C. The split ratio was 1:90. The oven temperature was programmed as follows: initial temperature was maintained at 45 °C for 4 min, then, heated to 280 °C at a temperature increasing rate of 3 °C/min.

At the end of each micropyrolysis, the residues were removed from the quartz tube and cleaned using the clean function of the micropyrolyzer (1000 °C for 5 s). After two micropyrolysis analyses, a blank analysis was performed (without biomass in the pyrolyzer) to clean the micropyrolyzer interface, the transfer line, and the separation column. Data processing was performed using the NIST library version 18, and only compounds with a similarity index (SI) greater than 80% were recorded.^26^

#### 2.3.2 Hydropyrolysis

Hydropyrolysis experiments were carried out for pure SSH and ex situ HZSM-5 zeolite in a mass ratio of 1:1. For each SSH sample passing through a 100 mesh sieve, analyses were performed in duplicate. Hydropyrolysis was also carried out using the CDS 5200 micropyrolyzer, using hydrogen 5.0 (99.999 purity) as the reactant gas and helium 5.0 (99.999 purity) as the carrier gas. The sample was positioned on the platinum resistance and inserted into the micropyrolyzer (see Figure 1). The heating rate and reaction temperature conditions were the same as those in fast pyrolysis.

After software initialization, the initial interface temperature was set to 75 °C and heated to 300 °C. Hydrogen was introduced during the interface temperature ramp. After the temperature reached 300 °C, the platinum resistor was rapidly heated to the reaction temperature at a heating rate of 20 °C/ms, and the reaction temperature was maintained for 10 s. Volatile products from hydropyrolysis were adsorbed on a Tenax adsorption column (heated to 65 °C) for 4 min. Then, the hydropyrolysis products adsorbed on the column were thermally desorbed at 300 °C for 1 min and dragged by helium to the GC/MS. The pyrolyzer/GC transfer line and pyrolyzer valve remained at 280 °C during all experiments.

The volatile products from the hydropyrolysis of each sample were analyzed using the GC/MS QP2010 Plus. The capillary column was the same as in the fast pyrolysis (Rtx-1701). Helium gas was the carrier gas with a column flow of 1 mL/min. The injector temperature was maintained at 250 °C, the interface temperature at 270 °C, and the ionization source temperature at 275 °C. The split ratio was 1:10. The oven temperature was programmed from an initial temperature of 45 °C for 4 min, then, heated to 280 °C at a heating rate of 3 °C/min.

Data processing was performed using NIST library version 18, and only compounds with a similarity index (SI) greater than 80% were recorded. Cleaning of the micropyrolyzer interface, transfer line, and separation column followed the same procedure previously described in fast pyrolysis.^26^

## 3 Results and Discussion

Tibola^28^ and Tibola et al.^29^ detailed the SSH sample characterization. In summary, the chemical composition (amount of lignin, holocellulose, and extractives), the moisture, volatile, ash, and fixed carbon contents, and the calorific value were considered typical for lignocellulosic biomass. Data of biomass chemical composition determined for SSH used in this work is presented in Table S1, in Supporting Information, and compared with other sources. Some divergences compared to other biomasses can be justified by the geographic location, the type of soil where the biomass was cultivated, and its intrinsic composition.

### 3.1 Analytical Micropyrolysis

The SSH samples were subjected to four analytical micropyrolysis conditions, namely: pure SSH in a helium atmosphere (CGHe), pure SSH in a hydrogen atmosphere (CGHi), SSH with HZSM-5 zeolite (ex situ) in a helium atmosphere (CG-ZeHe), and SSH with HZSM-5 zeolite (ex situ) in a hydrogen atmosphere (CG-ZeHi). The experiments were carried out at 450, 550, and 650 °C for each pyrolytic condition.

#### 3.1.1 Volatile Yields

**Figure 2:**
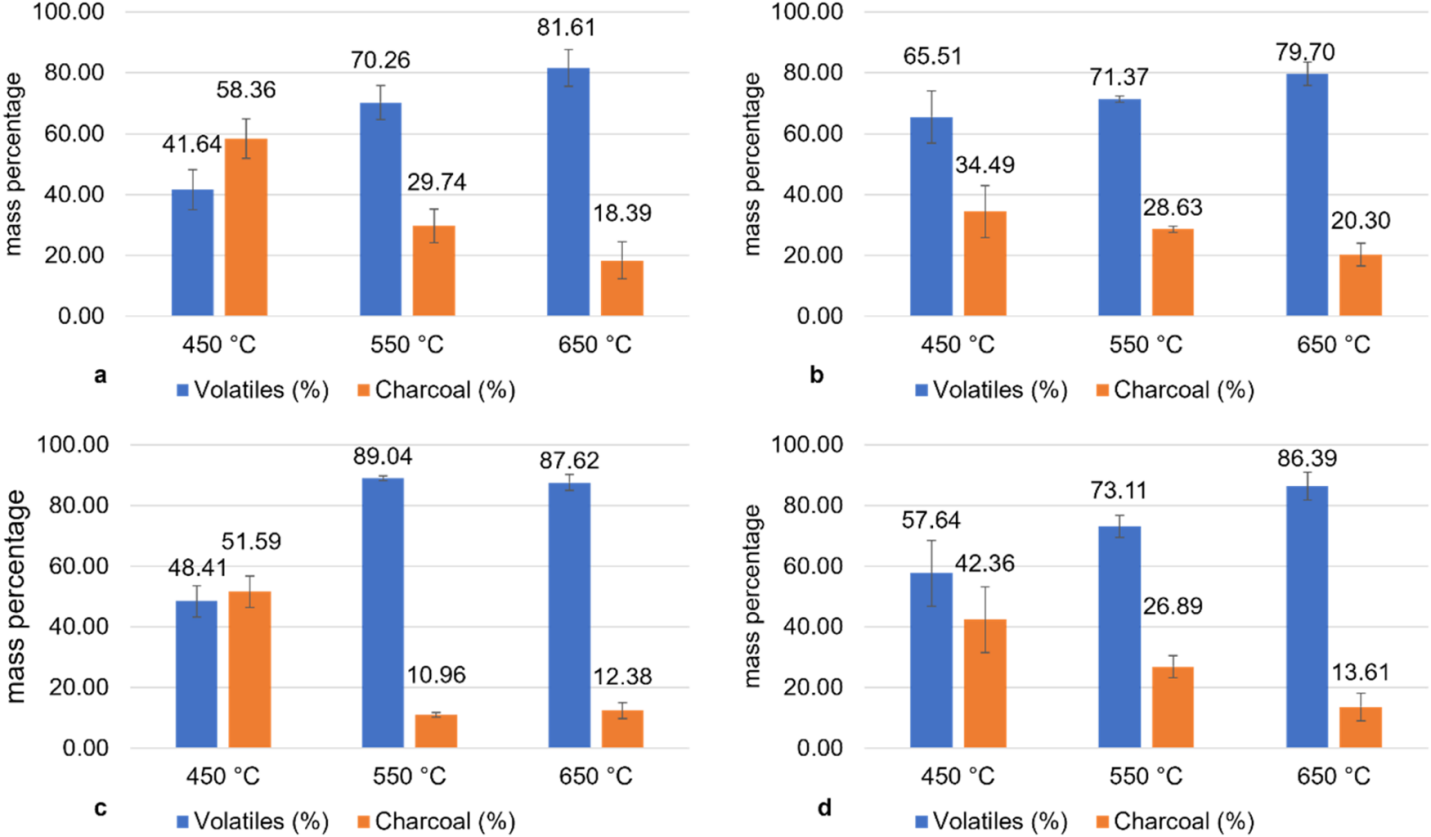
Fraction of volatiles and char produced by different pyrolytic conditions: (a) pure SSH in a helium atmosphere (CGHe); (b) pure SSH in a hydrogen atmosphere (CGHi); (c) SSH with HZSM-5 zeolite in a helium atmosphere (CG-ZeHe); and (d) SSH with HZSM-5 zeolite in a helium atmosphere (CG-ZeHi).

The volatile products and char yields, by mass, produced during the pyrolysis process can be determined by the difference between the initial biomass mass and the mass at the end of pyrolysis. Figure 2 presents the volatile products and char yields for each pyrolytic condition.

Figure 2a (CGHe) shows that the volatile products increased by increasing temperature from 42% at 450 °C to 82% at 650 °C. Consequently, the char decreased considerably by increasing temperature, reducing from 58% at 450 °C to 18% at 650 °C.

Figure 2b indicates that the increase in temperature led to higher yield of volatile products and a lower char yield for CGHi. The process at 650 °C was the most efficient, reaching volatile products of around 80%. However, at 450 °C, we can observe a significant difference compared to CGHe samples. The results for CGHe at 450 °C showed a higher yield for char (58%) instead of volatile products (42%). This indicates that, under a hydrogen atmosphere, all reaction temperatures favored higher production of volatile products than char. One possible explanation for this discrepancy between CGHi and CGHe results is that hydrogen at a lower temperature of 450 °C may have significantly assisted the primary thermal degradation of biomass, leading to an increased formation of volatile products.

Figure 2c shows that the fraction corresponding to volatile products is favored at higher temperatures. At 450 °C, the char yield was slightly higher than the volatile products, reaching 52% by mass. The volatile products yield at 550 and 650 °C was almost double compared to 450 °C, with an average of 88%, and the production of volatiles was slightly higher at 550 °C. The HZSM-5 zeolite favored the production of volatile products at all temperatures compared to CGHe, increasing their yields by 16, 27, and 7% for 450, 550, and 650 °C, respectively. This can be explained by the reaction capacity of the primary volatile products with the HZSM-5 zeolite active sites. The acidic sites of HZSM-5 zeolite likely helped the cracking of larger molecules and formation of volatile products while it also mitigated the repolymerization reactions that form char.^36,37^

Analyzing Figure 2d, the hydrogen atmosphere was more efficient for obtaining volatile products at 450 °C, with a 19% higher yield than CG-ZeHe, in Figure 2c. However, at 550 °C, the production of volatile products was 18% lower while, at 650 °C, both experimental configurations showed similar performances. This indicates that reducing hydrogen atmosphere at lower temperatures increases the production of volatile products, which is similar to CGHi experiments when compared to CGHe. At 550 °C, the HZSM-5 zeolite was more efficient under an inert atmosphere while, at 650 °C, the hydrogen atmosphere did not significantly affect it.

Studies have indicated that the highest yields of products obtained from fast pyrolysis of biomass are approximately 10–15% biochar and 85–90% volatile products, including bio-oil and permanent gases. Solid products (char) are favored at low temperatures because the extent of primary biomass degradation is still low. The char yield is similar at high temperatures since it can be gasified or thermally degraded by secondary reactions. The formation of bio-oil is maximized at 400–550 °C. This product can also undergo gasification above 600 °C due to secondary thermal degradation reactions.^36,38−40^ In this study, the higher yields for the volatile products at 650 °C may be predominantly due to primary thermal cracking since the residence time of the volatile products was short enough to avoid secondary reactions.

Onay^41^ investigated the fast pyrolysis of *Pistacia khinjuk* seeds in a fixed bed reactor and nitrogen atmosphere to produce bio-oil. The author observed an increase in the production of volatile products from 69 to 87% by increasing the final temperature from 400 to 700 °C. Lu et al.^42^ carried out analytical fast pyrolysis (Py-GC/MS) of various lignocellulosic biomasses using different catalysts to obtain high-quality bio-oil. The noncatalytic experiments in a helium atmosphere showed an increase in the production of volatile products by increasing temperature from 350 to 550 °C due to greater heat transfer and primary degradation reactions. Mourant et al.^43^ carried out fast pyrolysis of eucalyptus bark in a fluidized bed reactor to understand the effects of temperature on the bio-oil yields and composition. The authors observed that, under an inert atmosphere, the char yield decreased from 63 to 30% by weight by increasing temperature from 300 to 580 °C. Zhang et al.^44^ studied the catalytic fast pyrolysis of herbaceous biomass. The noncatalytic experiments showed that the total chromatographic peak area, representing the total yield of organic volatile products, increased by increasing pyrolysis temperature.

#### 3.1.2 CGHe Volatiles

**Figure 3:**
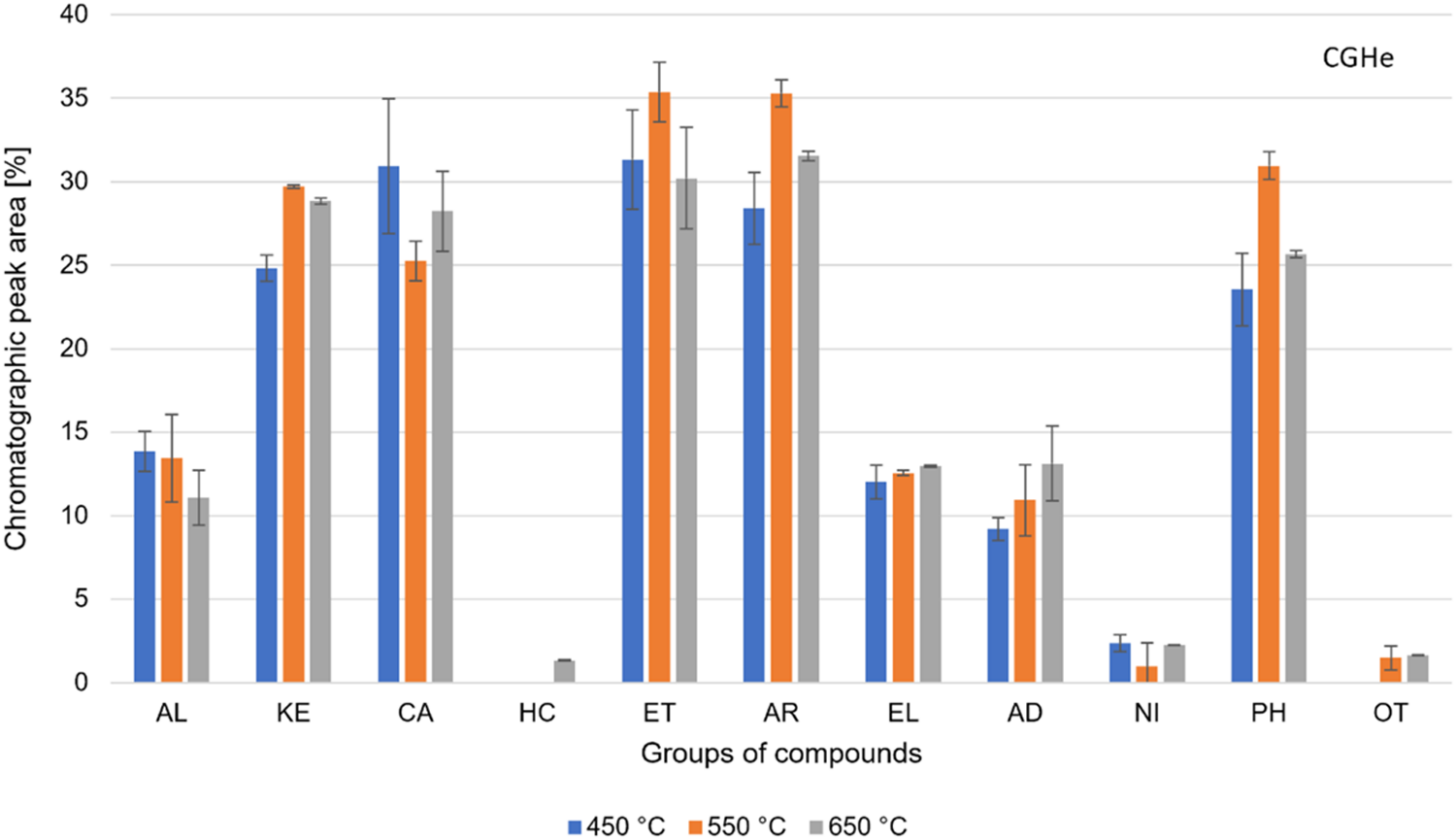
Groups of compounds present in the volatiles of CGHe pyrolysis. Alcohol (AL), ketones (KE), carboxylic acids (CA), hydrocarbons (HC), ethers (ET), aromatics (AR), esters/lactones (EL), aldehydes (AD), nitrogenous (NI), phenols (PH), and others (OT).

The Py-GC/MS system generated chromatograms for the chemical composition of the volatile products. The analysis of the volatile products is based on the % peak area. Although calibration with standards is necessary for a more accurate analysis, % peak area this measurement is considered linear with the component content. Figure 3 shows the chemical composition of the volatile products formed in the CGHe pyrolysis for each temperature (450, 550, and 650 °C). The organic compounds are classified and grouped according to their respective organic functions.

There was no standard behavior for the distribution of chemical groups by increasing temperature. The amount of alcohol (AL) slightly decreased by increasing temperature from 14% peak area at 450 °C to 11% at 650 °C. The esters/lactones (EL) and aldehydes (AD) groups showed a slight increase in % peak area with an average of 12.5% for EL and AD varying from 9% at 450 °C to 13% at 650 °C. Ketones (KE), carboxylic acids (CA), ethers (ET), aromatics (AR), and phenols (PH) groups showed small variations in their contents, mainly between 450 and 650 °C. For all groups, except CA, the highest % peak area values were at 550 °C, with KE and PH achieving approximately 30% and ET and AR 35%. The CA group presented a 31% peak area at 450 °C. The most chemical groups in the volatile products were KE, CA, ET, AR, and PH while AL, EL, and AD groups were the least present. In addition, there were no hydrocarbons (HC) in the volatile products, which suggests that almost all compounds are oxygenated.

The absence of HC and, consequently, the high presence of oxygen in the volatile products suggests that a bio-oil produced under these conditions would have undesirable properties to be applied as an alternative source of transport fuel. These properties include high acidity, chemical instability, and low calorific value, and they are mainly related to reactive oxygenated compounds, such as CA, AL, and KE.^45^ For an individual and semiquantitative analysis, Table 1 shows the main organic compounds in the CGHe volatile products at each temperature with their respective average values of % peak areas.

**Table 1:** Main Organic Compounds Present in CGHe Volatile Products

	organic compound	450 °C		550 °C		650 °C	
1	1-(4-hydroxy-3-methoxyphenyl)propan-2-one	1.03	±0.28	1.05	±0.09		
2	1,2-cyclopentanedione	2.60	±0.18	3.55	±0.37	3.57	±0.07
3	1-hydroxypropan-2-one	7.19	±0.12	9.21	±2.46	6.44	±0.19
4	2(5*H*)-furanone	0.84	±0.05	1.15	±0.11	1.19	±0.07
5	2,3-butanedione	2.60	±0.02	2.98	±0.35	2.84	±0.04
6	2,3-dihydro-1,4-dioxine	1.06	±0.04	0.39	±0.54		
7	2,6-dimethoxy-4-[(*E*)-prop-1-enyl]phenol	2.94	±0.42	2.31	±0.03	1.92	±0.06
8	2,6-dimethoxyphenol	1.82	±0.08	2.48	±0.18	1.76	±0.10
9	2-acetyloxyacetic acid			5.66	±0.21	6.79	±0.05
10	2-methoxy-4-[(*Z*)-prop-1-enyl]phenol	4.08	±0.64				
11	2-methoxy-4-vinylphenol	4.06	±0.62	4.51	±0.37	3.94	±0.17
12	2-methoxy-5-[(*E*)-prop-1-enyl]phenol					1.00	±0.06
13	2-methoxyphenol	2.17	±0.04	4.04	±0.16	3.81	±0.16
14	2-oxopropyl acetate	2.55	±0.45	1.88	±0.04	1.91	±0.10
15	3,5-dimethoxy-4-hydroxytoluene	0.49	±0.05	0.98	±0.40		
16	3-ethyl-2-hydroxycyclopent-2-en-1-one			1.80	±0.98	1.15	±0.04
17	3-methylcyclopentane-1,2-dione	1.11	±0.07	3.49	±0.73	2.72	±0.09
18	4-ethenyl-2,6-dimethoxyphenol	3.19	±0.20	3.18	±0.04	2.49	±0.10
19	acetic acid	27.00	±1.51	17.95	±0.94	16.96	±0.97
20	acetone					1.45	±0.08
21	creosol	0.25	±0.35	2.44	±0.30	2.25	±0.11
22	cyclopropyl carbinol	3.57	±0.26	2.57	±0.44	1.83	±0.01
23	d-allose			0.62	±0.88	1.85	±0.07
24	furfural	4.05	±0.23	4.35	±0.04	4.55	±0.04
25	methyl 2-oxopropanoate	1.78	±0.13	2.51	±0.18	3.08	±0.08
26	methyl acetate	5.52	±0.38				
27	methyl glyoxal	3.47	±0.01	1.85	±2.61	3.62	±0.25
28	oleic acid	3.56	±5.03	1.65	±2.33	3.98	±0.73
29	phenol	1.76	±0.17				
30	succindialdehyde			3.01	±0.23	2.54	±0.32
31	toluene					1.33	±0.04
32	*trans*-isoeugenol			3.46	±0.07	2.82	±0.09
33	vanillin	0.99	±0.09	1.17	±0.06	1.25	±0.06

From Table 1, we can observe that the compounds with the highest values of % peak area (above 5%) were almost the same for the temperatures studied. Acetic acid was the compound with the highest individual values at all temperatures and tended to lower yields by increasing temperature, varying from 27% at 450 °C to 17% at 650 °C. The 1-hydroxypropan-2-one was the second most abundant compound, with 9% peak area at 550 °C. The 2-acetyloxyacetic acid is not detected at 450 °C. However, at 650 °C, it showed 6.8% peak area. Methyl acetate, in turn, is detected only at 450 °C, with a 5.5% peak area.

Some phenolic compounds were significantly present in the composition of the volatile products. The 2-methoxy-4-vinylphenol was the most abundant phenolic compound in the volatile products and was detected at all temperatures with an average of 4.2% peak area. Another compound is furfural, an aromatic aldehyde present at all temperatures, with more than 4% peak area.

Most of these compounds in SSH volatile products come from the three main constituents of lignocellulosic biomass, i.e., cellulose, hemicelluloses, and lignin. Acetic acid is one of the main compounds and the most abundant organic acid in bio-oils from the fast pyrolysis of lignocellulosic biomass. It is mainly produced by the pyrolysis of holocellulose. In the pyrolysis of hemicellulose, the O-acetyl group can be released by xylose and ring opening of intermediates producing acetic acid. Cellulose undergoes depolymerization during pyrolysis to produce levoglucosan, and its fission produces acetic acid as a byproduct. Furthermore, this organic acid can be produced by further cracking of the acetyl group in the aliphatic chain of lignin components.^46^

The 1-hydroxypropan-2-one (acetol) is obtained from cellulose by the cleavage of the 1,4 glycosidic bond, intramolecular rearrangement of the monomeric units, and a cracking mechanism, which provides the remaining four-carbon fragments that are precursors in the formation of this compound through other rearrangement reactions.^47^ Furfural is mainly produced by the thermal degradation of hemicelluloses. However, it can also be produced by the thermal degradation of cellulose. In the pyrolysis of cellulose, its basic unit first breaks down, followed by ring opening, and dehydration, which leads to furfural. In hemicelluloses, xylan depolymerizes, and its branched chains are cleaved and removed along with the substituents, resulting in furfural.^48^ Phenolic compounds have aromatic rings substituted by hydroxyl. They are mainly derived from the decomposition of lignin. The pyrolysis of lignin includes three steps: exposure of phenolic hydroxyl groups, formation of monomer fragments, and removal of side chains. Due to the composite structure, various phenolic products can be generated from lignin. Thus, there is a strong structural correlation between lignin and phenolic compounds.^49^

Oliveira, Cardoso, and Ataíde^8^ also compared the products obtained from fast pyrolysis of soybean hulls in a fluidized bed reactor and analytical pyrolysis. Acetic acid presented the highest chromatographic peak area, especially at low temperatures. Tetradecane was another significant compound. The glyoxal consistently increased its peak area by increasing temperature. Some hydrocarbons only appeared at higher temperatures, such as benzene and toluene. Furfural was more present at low temperatures while phenolic compounds were barely detected.

Carvalho et al.^50^ performed fast pyrolysis of sweet sorghum bagasse in a fluidized bed unit and compared the results using analytical pyrolysis. The main compounds detected in the chromatograms were acetic acid, isoprene, methyl pyruvate, furfural, 2,3-dihydrobenzofuran, 4-hydroxy-3-methylacetophenone, and 5-hydroxymethylfurfural.

Casoni, Gutierrez, and Volpe^51^ studied the fast pyrolysis of SSH in a vertical reactor at 450 °C under an N_2_ atmosphere to investigate its bio-oil, which was analyzed by GC/MS. The main products were acetic acid (43%), furfural (12%), 2-methyl-4-propanol, 1,2-ethanediol diacetate, 2,3-butanedione (all 6%), 2-methoxyphenol (guaiacol) (5%), and smaller amounts of ketones, phenols, and acids. The authors observed that this bio-oil is highly unstable during storage since two phases are formed due to the repolymerization of methoxyphenols.

Bensidhom et al.^52^ performed fast pyrolysis of date palm seeds at different temperatures. Considering chromatographic peak area, the main compounds obtained were anhydrous sugars (up to 23% at 450 °C), acids (up to 20% at 500 °C), ketones (up to 17% at 600 °C), furans (up to 14% at 500 °C), phenols (up to 4% at 500 °C), and many other compounds, such as aldehydes, alcohols, ethers, pyran derivatives, hydrocarbons, and compounds containing N and S.

David et al.^53^ detailed the composition characteristics of volatile products from the pyrolysis of pecan shells using Py-GC/MS. The organic compounds identified in the volatile products showed that oxygenated compounds had their relative concentration decreased by increasing temperature from 550 to 650 °C. The polar oxygenated groups presented their highest concentrations at 450 °C, and the most abundant compounds were carboxylic acids (34.03%), phenols (10.18%), ketones (10.05%), aldehydes (5.42%), and esters (4.34%). The furan content decreased from 3.57 to 1.83% when the final pyrolysis temperature increased from 450 to 650 °C. The concentration of hydrocarbons increased by increasing temperature, achieving its maximum values at 650 °C, both for aliphatic (58.93%) and aromatic (5.46%) hydrocarbons.

#### 3.1.3 CGHi Volatiles

**Figure 4:**
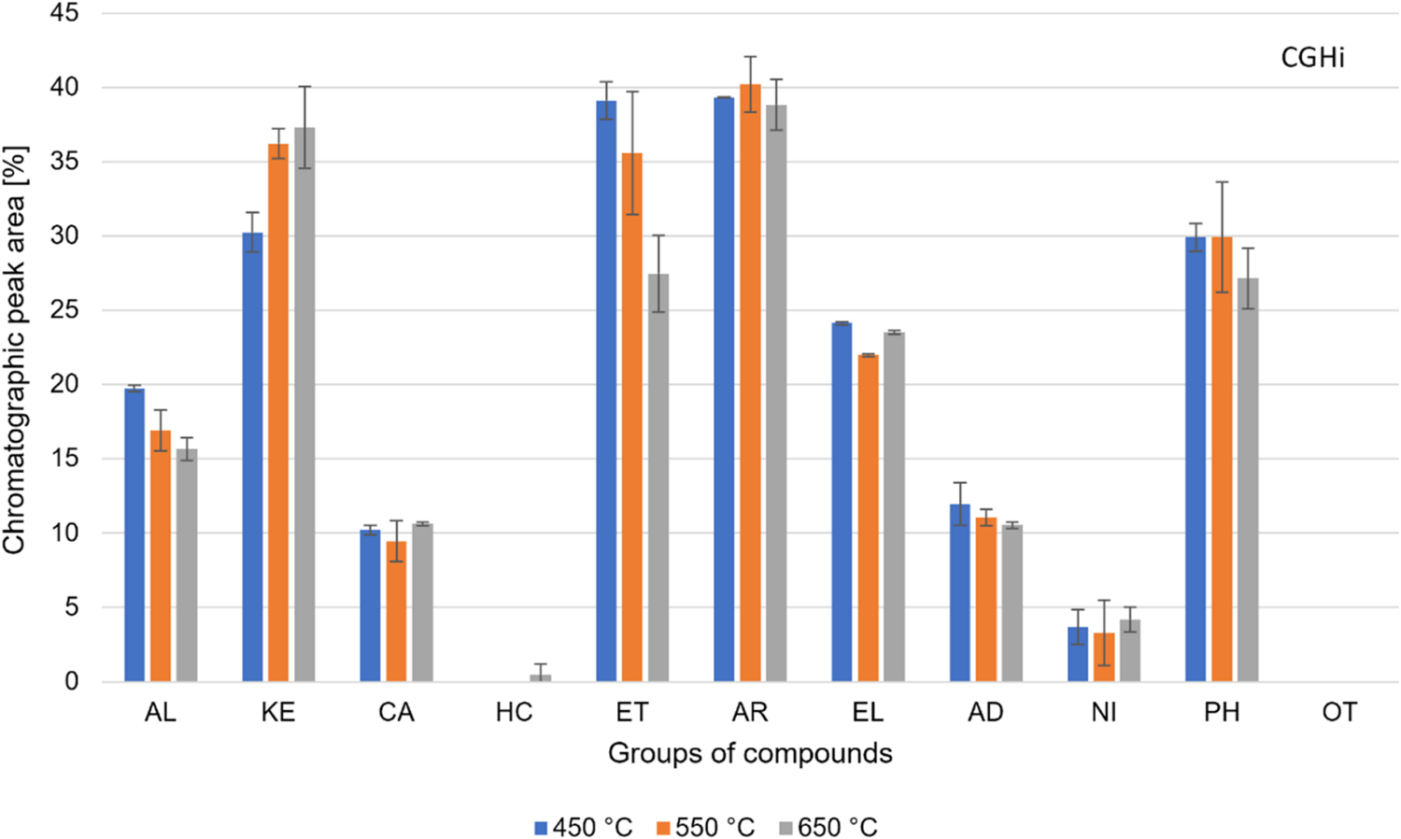
Groups of compounds present in the volatile products of the CGHi pyrolysis. Alcohol (AL), ketones (KE), carboxylic acids (CA), hydrocarbons (HC), ethers (ET), aromatics (AR), esters/lactones (EL), aldehydes (AD), nitrogenous (NI), phenols (PH), and others (OT).

The analysis of the volatile products formed during the CGHi pyrolysis carried out in the Py-GC/MS system is based on the % peak area. Figure 4 shows the composition of the volatile products from the CGHi pyrolysis for each temperature. The organic compounds are grouped and classified according to their respective organic functions.

The results did not show any standard behavior for the distribution of organic groups by increasing temperature. The % peak areas of AL, ET, AD, and PH groups decreased by increasing temperature. The ET group presented the most significant reduction, varying from 39% peak area at 450 °C to 27% at 650 °C. The AL group presented its highest % peak area at 450 °C (20%). The PH group showed 27% peak area at 650 °C.

The KE group was the only one that significantly increased % peak area by increasing temperature, varying from 30% at 450 °C to 37% at 650 °C. For CA, AR, EL, and NI groups, the increase in temperature did not significantly change the % peak area. The CA, EL, and NI groups presented an average of 10, 23, and 4% peak area, respectively. The AR group showed the highest % peak area, reaching an average of 39%.

Comparing the distribution of organic groups in CGHi and CGHe experiments, we can observe that the AL, KE, AR, EL, and NI groups presented a higher % peak area under a hydrogen atmosphere for all temperatures. The ET and PH groups increased the % peak area at 450 °C. The CA group showed a significant drop in the % peak area at all temperatures. However, both CGHi and CGHe experiments did not produce HC.

Despite the reactive hydrogen atmosphere, volatile organic compounds are not deoxygenated. According to some studies, this may be due to effective deoxygenation requires higher hydrogen pressures (around 30 bar) in which there are sufficient reactive hydrogen radicals to convert oxygenated compounds into hydrocarbons and water.^9,54^

Dayton et al.^55^ studied the fast hydropyrolysis of residual biomass in a pressurized fluidized bed reactor. The authors showed almost no deoxygenation at 400 °C when using inert material in the bed and a low hydrogen partial pressure (0.5–3.0 bar). The resulting bio-oil contained 35–39 wt % oxygen compared to 38–40 wt % oxygen after fast pyrolysis in a nitrogen atmosphere.

Jan et al.^56^ investigated the catalytic fast hydropyrolysis of Poplar lignin to produce cycloalkanes. The experiments analyzed the effect of catalyst/lignin ratio, hydrogen partial pressure, and temperature on hydrocarbon yields. The experiments without a catalyst and at atmospheric pressure showed the same reaction products as those from the pyrolysis in an inert atmosphere (He), with phenolic compounds as the primary products and only traces of aromatic hydrocarbons.

Machado et al.^54^ carried out fast hydropyrolysis of Canadian pine wood under H_2_ flow at atmospheric pressure and 500 °C. The volatile products were analyzed on a GC/MS system and showed the formation of large amounts of oxygenated products, including 9% acids, 38% carbonyl-containing compounds, 12% furans, and 28% phenols.

Venkatesan et al.^57^ studied the production of aromatic hydrocarbons through fast hydropyrolysis of biomass followed by catalytic upgrading at 500 °C on a Py-GC/MS system. The authors compared the fast pyrolysis of Pine wood in an inert atmosphere with fast hydropyrolysis. The results showed similar trends for both reaction conditions, with the fast hydropyrolysis producing 28% phenols, 50% oxygenates, and 16% furans. They concluded that there was no effect of hydrogen gas on the composition of the pyrolysis products in the absence of a catalyst.

Hu et al.^37^ investigated catalytic fast hydropyrolysis of seaweed to produce liquid fuels. They analyzed the effect of hydrogen compared to nitrogen in a fixed bed and the effect of hydrogen with catalysts on the functional groups and components in the bio-oil. The results indicated an insignificant effect of hydrogen in the absence of the catalyst. In the hydrogen experiments without a catalyst, anhydro sugars and phenols decreased and alcohols increased while N-containing substances, chain hydrocarbons, aromatics, furans, small molecular weight aldehydes, and ketones remained almost unchanged compared to nitrogen experiments. Table 2 shows the main organic compounds identified in the CGHi volatile products at each temperature with their respective average values of % peak areas.

**Table 2:** Main Organic Compounds Present in CGHi Volatile Products

	organic compound	450 °C		550 °C		650 °C	
1	1-(4-hydroxy-3-methoxyphenyl)propan-2-one	1.91	±0.22	1.88	±0.25	1.60	±0.11
2	1-(furan-2-yl)ethanone	0.84	±0.09	1.41	±0.04	1.85	±0.40
3	1,2-cyclopentanedione	5.17	±0.01	4.58	±0.02	4.44	±0.18
4	1-hydroxypropan-2-one	8.69	±0.39	9.73	±0.45	10.43	±0.58
5	2(5*H*)-furanone	3.23	±0.06	3.49	±0.05	3.08	±0.17
6	2,3-butanedione	1.61	±0.32				
7	2,3-dihydro-1,4-dioxine	2.09	±0.00	1.32	±0.01	1.29	±0.21
8	2,6-dimethoxy-4-prop-2-enylphenol	3.19	±0.58	2.35	±0.53		
9	2,6-dimethoxyphenol	2.52	±0.08	2.15	±0.28	1.58	±0.10
10	2-acetyloxyacetic acid	9.33	±0.18	8.41	±0.11	10.62	±0.12
11	2-butenal					1.36	±0.15
12	2-furanmethanol	2.27	±0.04	1.22	±1.72	2.42	±0.21
13	2-hydroxy-3-methylcyclopent-2-en-1-one	2.87	±0.23	5.00	±0.16	4.73	±0.25
14	2-methoxy-4-[(*Z)*-prop-1-enyl]phenol	1.48	±0.01				
15	2-methoxy-4-vinylphenol	3.10	±0.35	2.57	±1.41	1.99	±0.67
16	2-methoxy-5-methylphenol			2.80	±0.00	2.85	±0.15
17	2-methoxyphenol	2.65	±0.21	4.20	±0.81	4.76	±0.37
18	2-methylphenol					1.88	±0.07
19	2-oxopropyl acetate	3.16	±0.07	3.23	±0.08	3.42	±0.15
20	4-cyclopentene-1,3-dione	1.19	±0.07	1.37	±0.03	1.67	±0.17
21	4-ethenyl-2,6-dimethoxyphenol	3.23	±0.08	2.62	±0.00	2.10	±0.02
22	4-ethyl-3,4-dihydro-2*H*-pyrrole			0.60	±0.84	1.58	±0.45
23	4-hydroxybutanoic acid	0.88	±0.12				
24	creosol	2.98	±2.75				
25	cyclopropyl carbinol	6.45	±0.01	3.53	±0.12	2.81	±0.01
26	eugenol	2.82	±2.02	2.08	±0.30	1.65	±0.08
27	furfural	5.70	±0.01	5.67	±0.25	6.22	±0.04
28	methyl 2-oxopropanoate	4.17	±0.08	5.56	±0.01	5.98	±0.28
29	*p*-cresol					2.55	±0.25
30	phenol	2.55	±0.34	4.28	±0.33	4.62	±0.01
31	propanal	3.39	±0.28				
32	vanillin	2.11	±0.06	2.00	±0.03		

The results in Table 2 indicate that 1-hydroxy-2-propanone (acetol) and 2-acetyloxyacetic acid were the compounds with the highest yield at all temperatures. Furfural showed a similar trend, except at 450 °C. The increase in temperature did not significantly change the production of these compounds. However, they presented their highest % peak areas at the highest temperature (650 °C), achieving 10.62, 10.43, and 6.22% for 2-acetyloxyacetic acid, acetol, and furfural, respectively.

At 450 °C, 1,2-cyclopentanedione and cyclopropyl methanol presented 5.17 and 6.45% peak area, respectively. Both compounds decreased by increasing temperature. At 550 °C, 2-hydroxy-3-methylcyclopent-2-en-1-one (cyclooctene) showed 5% peak area and lower values at extreme temperatures (450 and 650 °C). The methyl 2-oxopropanoate (methyl pyruvate) was also significant in the volatile products, and increased by increasing temperature, with a 5.98% peak area at 650 °C.

Cyclooctene, 1,2-cyclopentanedione, and cyclopropyl methanol are probably originated from cellulose through reactions such as depolymerization, cracking, ring opening, and rearrangement. Cyclooctene and 1,2-cyclopentanedione have antioxidative properties. Cyclopropyl methanol is considered a dangerous compound since it is flammable, corrosive, irritating, and harmful to health. However, these compounds can be applied to pharmacology and medicine and as intermediates in organic synthesis. Furthermore, cyclooctene is a food flavoring and can be used to provide coffee and caramel aromas in the food industry.^58−60^ Methyl pyruvate can be obtained mainly from reactions involving acetyl groups of lignocelluloses. It is a chemical product with high value-added and is widely used as an intermediate for the synthesis of perfumes, polymer industry, food additives, amino acids, agrochemicals, and drugs.^61^

#### 3.1.4 CG-ZeHe Volatiles

**Figure 5:**
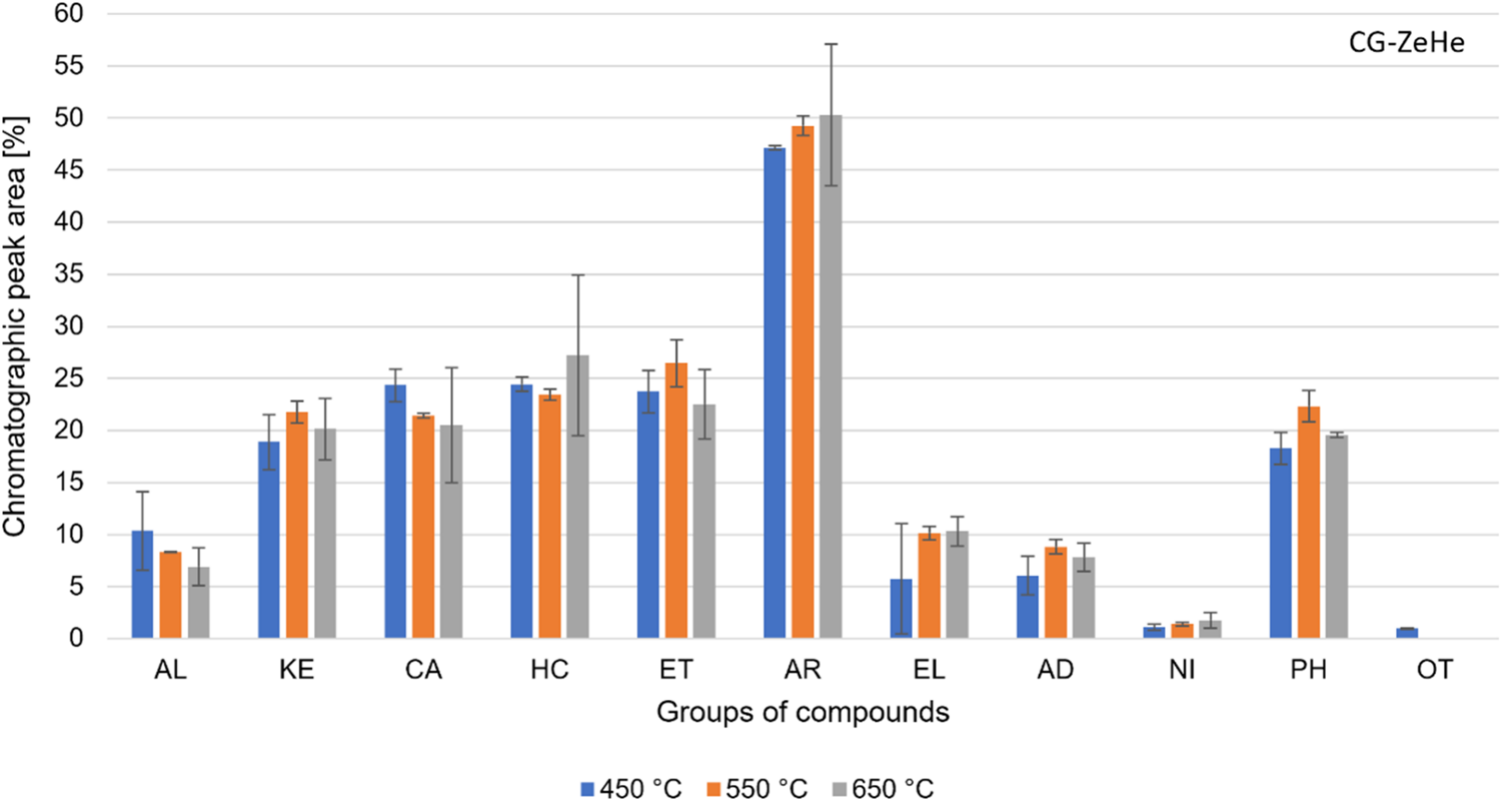
Groups of compounds present in the volatile products of CG-ZeHe pyrolysis. Alcohol (AL), ketones (KE), carboxylic acids (CA), hydrocarbons (HC), ethers (ET), aromatics (AR), esters/lactones (EL), aldehydes (AD), nitrogenous (NI), phenols (PH), and others (OT).

The analysis of the volatile products formed during the CG-ZeHe pyrolysis carried out in the Py-GC/MS system is based on the % peak area. Figure 5 shows the composition of the volatile products formed during the CG-ZeHe pyrolysis at each temperature. The compounds are grouped and classified according to their respective organic functions.

Figure 5 shows that temperature did not significantly change the yield of the organic compounds in the volatile products. However, the AL and CA groups decreased by increasing temperature, presenting an average of 9 and 22% peak area, respectively. The AR and EL groups increased by increasing temperature, achieving an average of 49 and 9% peak area, respectively. The highest values for KE, ET, AD, and PH groups were 22, 26, 9, and 22% peak area, respectively, at 550 °C.

The CG-ZeHe experiments compared to the CGHe showed that the HZSM-5 zeolite promoted, to a certain extent, deoxygenation of the volatile products. This is highlighted by the presence of HC in the Py-GC/MS system. Comparing Figure 3 with Figure 5, the HZSM-5 zeolite catalyst decreased in the % peak area for almost all organic groups, except for HC and AR. Furthermore, the HC group was detected at all temperatures, and temperature did not significantly influence CG-ZeHe. The HC group achieved an average of 25% peak area, which is much higher than the CGHe experiments in which HC was not produced.

The formation of HC indicates that a bio-oil obtained from CG-ZeHe would have a reduced oxygenate content, probably changing its properties to lower acidity and viscosity, in addition to greater chemical stability and energy content. These properties may be desirable in high-quality liquid fuels and high-value-added chemicals.^36,48^ In catalytic fast pyrolysis with HZSM-5, intermediate volatile compounds, produced from the primary pyrolysis of biomass, come into contact with the zeolite and undergo cracking reactions (dehydration, decarbonylation, decarboxylation, and aromatization) in the acidic sites and, release oxygen as H_2_O and CO_*x*_ (CO and CO_2_) to form HC.^48,62^

The primary anhydrous sugars from the degradation of cellulose and hemicelluloses react further with lighter furan and oxygenated compounds. These can create a so-called “pool” of hydrocarbons, which is described as a catalytic site composed of larger HC (generally aromatics and olefins) adsorbed in the micropores of HZSM-5 in which cracking reactions occur in a cycle, leading to the production of monoaromatics and light olefins. Then, monoaromatics can further oligomerize, possibly under the incorporation of light olefins, to polycyclic aromatics and, eventually, coke. Highly oxygenated aromatics derived from lignin are mainly converted into simple phenolic compounds based on the high dissociation energy required to break the phenolic CO bond (466 kJ/mol). However, if the phenolic bond breaks, e.g., in strong acidic sites and/or at high reaction temperature, monoaromatics are formed.^63,64^Table 3 shows the main organic compounds identified in the CG-ZeHe volatile products at each temperature with their respective average values of % peak areas.

**Table 3:** Main Organic Compounds Present in CG-ZeHe Volatile Products

	organic compound	450 °C		550 °C		650 °C	
1	(*E*)-2,6-dimethoxy-4-(prop-1-en-1-yl)phenol	1.32	±1.87	2.08	±0.82		
2	(*E*)-octadec-9-enoic acid					5.52	±0.08
3	1,2,3-trimethylbenzene	2.92	±0.18	0.91	±1.29	1.50	±0.47
4	1,2-cyclopentanedione	2.17	±0.48	3.50	±0.21	3.11	±0.43
5	1-ethyl-3-methylbenzene	1.93	±0.34			0.60	±0.85
6	1-hydroxypropan-2-one	6.20	±2.32	5.55	±0.31	4.63	±0.84
7	1-methylnaphthalene	0.90	±0.29	2.10	±0.04	1.02	±1.44
8	2(5*H*)-furanone	0.66	±0.07	1.07	±0.13	1.33	±0.31
9	2,2-diethyl-3-methyl-1,3-oxazolidine	1.10	±0.26	1.40	±0.21	0.97	±0.37
10	2,3-butanedione	2.05	±0.06	1.72	±0.11	1.73	±0.23
11	2,6-dimethoxyphenol	1.56	±0.06	1.93	±0.28	1.59	±0.04
12	2-acetyloxyacetic acid			5.41	±0.02	2.96	±4.19
13	2-methoxy-4-vinylphenol	3.55	±0.23	4.00	±0.08	3.55	±0.30
14	2-methoxyphenol	2.15	±0.28	4.06	±0.04	3.56	±0.16
15	2-oxopropyl acetate	0.89	±1.25	1.63	±0.48	1.43	±0.08
16	3-methylcyclopentane-1,2-dione	0.90	±0.05	2.61	±0.26	2.04	±0.33
17	4-ethenyl-2,6-dimethoxyphenol	2.63	±0.08	2.84	±0.27	2.27	±0.13
18	acetic acid	22.26	±4.53	12.60	±0.06	12.05	±1.40
19	acetone			0.37	±0.52	1.11	±0.04
20	benzene	1.44	±0.19	2.47	±0.11	3.37	±0.62
21	creosol	0.30	±0.42	1.63	±0.05	1.92	±0.22
22	cyclopropyl carbinol	2.90	±0.33	2.38	±0.25	0.90	±1.27
23	furfural	2.69	±0.05	3.51	±0.07	3.51	±0.64
24	methyl 2-oxopropanoate	1.27	±0.00	2.05	±0.03	2.13	±0.48
25	methylglyoxal	1.65	±2.33	2.57	±0.03	2.28	±0.52
26	naphthalene			1.40	±0.21	2.34	±0.64
27	oleic acid	2.10	±2.96	3.42	±0.26		
28	*o*-xylene	10.06	±0.04	2.41	±0.06	2.29	±0.49
29	phenol	0.75	±1.06	1.16	±0.05	1.55	±0.46
30	*p*-xylene			6.21	±0.01	6.06	±0.84
31	succindialdehyde			2.32	±0.02	2.02	±0.21
32	toluene	6.03	±0.26	6.21	±0.13	6.80	±0.61
33	trans-isoeugenol			2.86	±0.16	2.57	±0.15

Acetic acid showed the highest % peak area for all temperatures, achieving a 22% peak area at 450 °C. At higher temperatures, it presented similar % peak area values and a decrease of approximately 50%. This behavior can also be observed in the CGHe experiments (see Table 1). However, in the CG-ZeHe experiments, the % peak area values were lower may be due to the performance of the HZSM-5 zeolite. Toluene was another compound with a high % peak area at all temperatures. However, the increase in temperature significantly increased its yield, with an average of 6% peak area. The 1,2-xylene (*o*-xylene) presented a maximum of 10% peak area at 450 °C. The 1,4-xylene (*p*-xylene) presented an average of 6% peak area at higher temperatures, but it was not detected at 450 °C. Acetol was identified at all temperatures but decreased from 6.2 to 4.6% peak area by increasing temperature. The 2-acetyloxyacetic acid was formed only at the highest temperatures, presenting the highest value of 5.4% peak area at 550 °C. The (*E*)-octadec-9-enoic acid (elaidic acid) showed a 5.5% peak area at 650 °C, but it was not detected at lower temperatures.

The production of aromatic hydrocarbons (HCAr) from biomass derivatives has great importance in establishing a renewable source of these compounds since they still mainly come from petroleum.^65^ We detected these products in the CG-ZeHe experiments in which monocyclic HCAr, such as benzene, toluene, and xylenes (BTX), were the main compounds. Polycyclic HCAr, such as naphthalene, was produced in smaller quantities.

The BTX are the main chemicals in the light aromatic group, known as the premium organic building blocks in the chemical industry. These products are highly valuable and can be applied in different areas. Specifically, benzene is mainly used as a precursor to styrene, phenol, nylon, and aniline. Toluene is normally mixed with unleaded gasoline due to its low toxicity and high octane number. Most of the toluene produced is directly converted into benzene and xylenes, in addition to solvent applications. O-xylene is converted into phthalic anhydride. The *p*-xylene is the most valuable xylene isomer and is converted into terephthalic acid and dimethyl terephthalate, which are used to produce polyethylene terephthalate (PET) fibers, resins, and films.^66,67^

The liquid chromatography technique is effective to isolate HCAr in the fast pyrolysis crude bio-oil. However, the high consumption of solvents and the regeneration of the solid phase of silica gel make the process more expensive. Steam distillation is an interesting alternative since the characteristics of bio-oil make it thermosensitive, and this method can reach boiling temperatures lower than the normal boiling points of compounds by adding water vapor and separating HCAr. Studies have shown that a less oxygenated bio-oil, i.e., more stable and less acidic, obtained by catalytic fast pyrolysis using zeolites, for example, can have its BTX fractions isolated through conventional fractional distillation.^68,69^

Promsampao et al.^70^ analyzed the effect of hydrothermal treatment on HZSM-5 on the production of bio-oil via ex situ fast pyrolysis of eucalyptus wood. Pyrolysis was carried out in a fluidized bed reactor at 500 °C, in nitrogen flow, and varying biomass to catalyst (B/C) ratios. The authors used a GC/MS system to determine the bio-oil composition. The hydrothermal treatment increased approximately 30% of the organic liquid yield for all applied B/C ratios, reaching a maximum yield of 11.4% by weight, based on dry biomass, for the lowest B/C ratio of 0.4. This organic liquid had 5.9% by weight of oxygen and contained mainly HCAr, polycyclic (two-ring), and monocyclic. The catalytic bio-oils produced were deeply deoxygenated with a maximum degree of deoxygenation of 93%.

Toro-Trochez et al.^71^ compared fast pyrolysis and catalytic fast pyrolysis of soybean hulls. The experiments were carried out at temperatures ranging from 400 to 600 °C, in a nitrogen atmosphere and with the HZSM-5 zeolite. The fast pyrolysis and catalytic fast pyrolysis achieved similar bio-oil yields (38–45 and 37–42%, respectively). Phenolic compounds increased by more than 16% after the catalytic fast pyrolysis. Furthermore, HC increased from 5 to 9% while CE decreased by about 37–49%. This led to a 14–19% decrease in the number of total acids and an increase in energy content of around 30% after catalytic fast pyrolysis. The biogas yield increased in catalytic fast pyrolysis due to the deoxygenation and cracking reaction catalyzed by HZSM-5 zeolite, and its energy content was 15.6 MJ/mol.

Socci et al.^72^ studied the effect of acidity and shape selectivity of different catalysts on the catalytic fast pyrolysis of beech wood at 500 °C in a helium atmosphere. The volatile products were analyzed by GC/MS. Total gas production increased approximately 3-fold using ZSM-5 catalyst at a C/B ratio of 1:1 compared to noncatalytic pyrolysis. Furthermore, the ZSM-5 catalyst was particularly effective in producing monocyclic HCAr. At this same C/B ratio (1:1), the HCAr yield was 2 C% and increased significantly to almost 14 C% in a 5:1 ratio. The authors highlighted the importance of catalyst shape selectivity to convert pyrolysis volatile products into desirable products.

Guo et al.^73^ performed the catalytic fast pyrolysis of *Arundo donax* (giant sugar cane) using modified HZSM-5 zeolite catalysts in a two-stage fixed bed reactor at 500 °C in a nitrogen atmosphere to investigate the catalytic performance. The results showed that the addition of unmodified HZSM-5 zeolite substantially decreased the total liquid yield from 49.53% to 43.53 wt % while improving the formation of gaseous products from 24.90 to 30.79% by weight compared to noncatalytic experiments. Furthermore, undesired oxygenates decreased from 62.66 to 21.82% peak area while improving the selectivity for HCAr from approximately 2% peak area without a catalyst to 25% with HZSM-5 zeolite.

Li et al.^74^ investigated the catalytic effects of Fe- and Zn-modified HZSM-5 with different mass ratios, loading rates, and biomass/catalyst ratios on aromatics selectivity. Catalytic fast pyrolysis of bamboo waste was carried out at 500 °C, in a helium atmosphere, and using Py-GC/MS analysis. The % peak area of several oxygenated compounds decreased by adding HZSM-5. Aromatics were identified only after the addition of the catalyst. Bimetallic HZSM-5 was superior to unmodified HZSM-5 for selectivity and monocyclic HCAr yield. When the mass ratio of Fe to Zn was 4:1 at a loading ratio of 5 wt % and the mass ratio of catalyst to biomass was 2:1, the best deoxidation capacity and the highest HCAr yield (93.8 mg/g), especially for toluene (28.0 mg/g) and xylene (27.2 mg/g), was achieved compared to the other catalytic configurations.

#### 3.1.5 CG-ZeHi Volatiles

**Figure 6:**
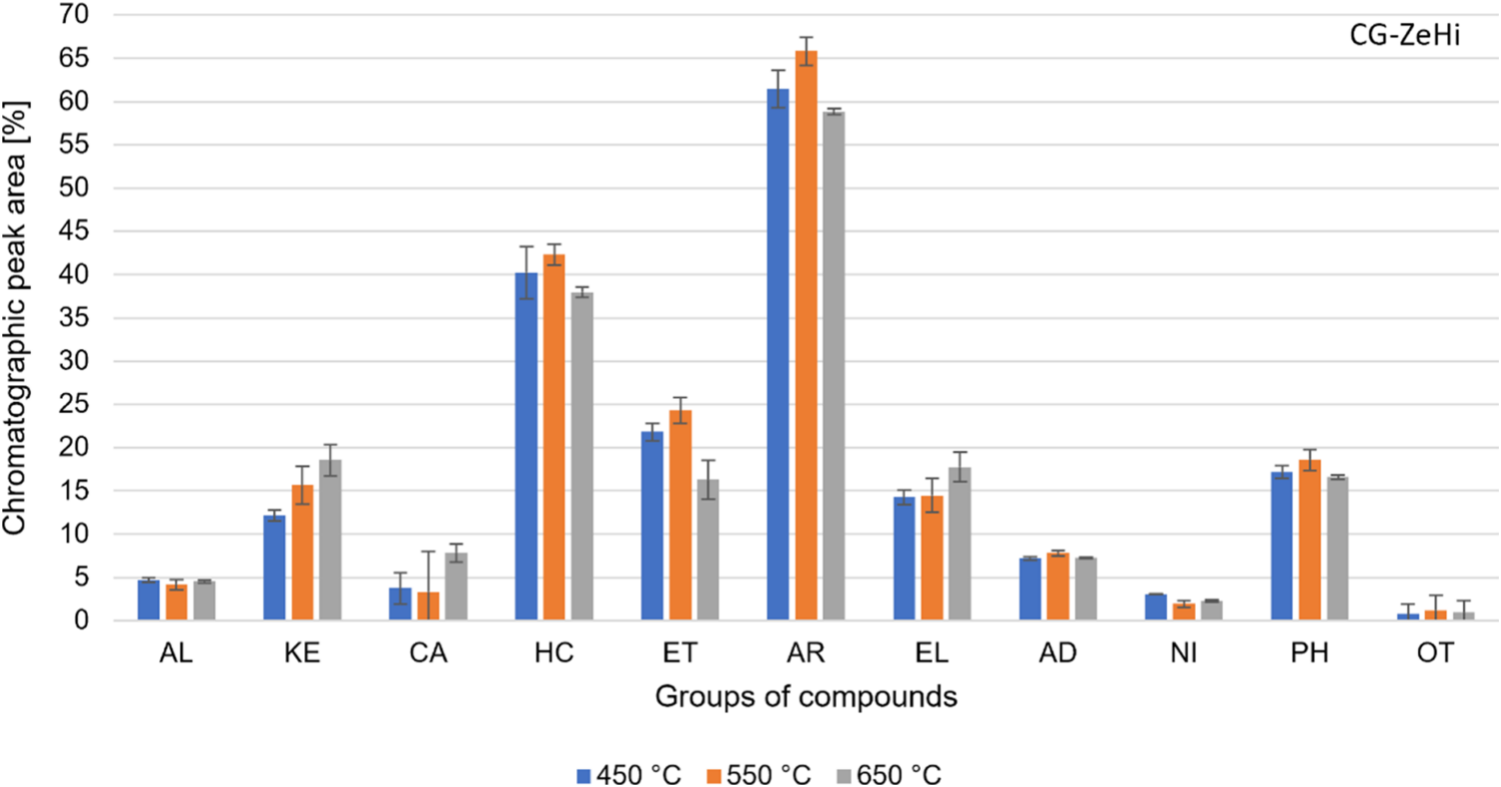
Groups of compounds present in the volatiles of CG-ZeHi pyrolysis. Alcohol (AL), ketones (KE), carboxylic acids (CA), hydrocarbons (HC), ethers (ET), aromatics (AR), esters/lactones (EL), aldehydes (AD), nitrogenous (NI), phenols (PH), and others (OT)

The analysis of the volatile compounds formed during the CG-ZeHi pyrolysis carried out in the Py-GC/MS system is based on the % peak area. Figure 6 shows the composition of the volatile products formed by the CG-ZeHi pyrolysis for each temperature. The organic compounds are grouped and classified according to their respective organic functions.

These results did not show any standard behavior for the distribution of organic functions as a function of the temperature. The KE, CA, and EL groups increased by increasing temperature. The CA group doubled from 4 to 8% peak area while KE and EL groups showed 19 and 18% peak area, respectively, at 650 °C. The ET, HC, and AR groups achieved maximum values at 550 °C, and AR presented the highest % peak area (66%) among all compounds. The AL, AD, NI, and PH groups showed little variation, with averages of 4, 7, 2, and 17% peak areas, respectively.

Compared to the CG-ZeHe experiments (see Figure 5), the CG-ZeHi experiments changed the average distribution of organic functions in the volatile products, especially for HC, EL, and CA groups. For HC and EL groups, the CG-ZeHi experiments showed higher % peak area of 60% and 77%, respectively, while the CA group reduced by 78%. The content of other organic compounds also changed but to a lesser extent. The AL, KE, ET, and PH groups decreased by 48, 24, 14, and 13%, respectively, and the AR group increased by 27% peak area. The amount of HC obtained for oxygenated compounds was significantly higher than all other experimental configurations. The increase in temperature did not significantly affect HC production, achieving a maximum of 42% peak area at 550 °C.

These results suggest that the HZSM-5 zeolite in a hydrogen atmosphere favored the deoxygenation reactions (hydrodeoxygenation, decarboxylation, decarbonylation, aromatization, cracking, rearrangement) to produce HC. Regarding the general mechanisms by which deoxygenation reactions occur, the only difference compared to the previously mentioned mechanisms for the CG-ZeHe experiments is the reactive atmosphere. In the presence of hydrogen gas, more H radicals may have been produced to interact with reactive primary organic volatile products in the acidic sites of the zeolite. These interactions contributed to deoxygenation reactions. Decarboxylation reactions were important for this process, given the sharp drop in yield observed for carboxylic acids. Table 4 presents the main organic compounds present in CG-ZeHi pyrolysis volatiles at each reaction temperature with their respective average values of % peak areas.

**Table 4:** Main Organic Compounds Present in CG-ZeHi Volatiles

	organic compound	450 °C		550 °C		650 °C	
1	1,2-cyclopentanedione	3.37	±0.31	4.89	±0.20	4.85	±0.10
2	1,3-xylene	11.02	±0.52	6.48	±0.54	6.22	±0.06
3	1-ethyl-2-methylbenzene	4.28	±0.20	3.14	±0.31	2.69	±0.17
4	2(5*H*)-furanone	2.00	±0.09	2.38	±0.30	3.99	±0.17
5	2,2-diethyl-3-methyl-1,3-oxazolidine	3.05	±0.06	0.85	±1.20		
6	2,3-butanedione	2.09	±0.14				
7	2,6-dimethoxyphenol	1.69	±0.01	3.33	±0.05	0.97	±1.37
8	2-acetyloxyacetic acid			3.33	±4.70	7.12	±0.09
9	2-hydroxy-3-methylcyclopent-2-en-1-one	0.90	±1.27			3.80	±0.20
10	2-methoxy-4-vinylphenol	1.46	±2.06	2.19	±0.21	1.81	±0.42
11	2-methoxy-5-methylphenol			2.77	±0.88	2.18	±0.16
12	2-methoxyphenol	3.16	±0.02	4.11	±0.18	4.08	±0.28
13	2-methylnaphthalene	1.76	±0.10	2.26	±0.11	2.04	±0.13
14	2-oxopropyl acetate	2.03	±0.05	2.06	±0.15	2.30	±0.06
15	3-furaldehyde	4.08	±0.05				
16	3-methylcyclopentane-1,2-dione	0.93	±1.32	3.65	±0.30		
17	4-ethenyl-2,6-dimethoxyphenol	2.47	±0.08	1.90	±0.06	1.49	±0.25
18	acetic acid	2.88	±0.57				
19	benzene	3.51	±0.27	4.00	±0.33	3.67	±0.18
20	cyclopropyl carbinol	3.54	±0.15	3.60	±0.25	3.09	±0.18
21	ethyl 2-acetyloxyacetate	6.94	±0.16	3.24	±4.58		
22	furfural			3.72	±0.13	4.27	±0.08
23	Indane	2.26	±0.91	2.95	±0.38	2.56	±0.11
24	mesitylene	4.27	±0.14	3.46	±0.10	1.59	±2.24
25	methyl 2-oxopropanoate	2.00	±0.30	2.82	±0.49	3.49	±0.19
26	naphthalene			1.56	±0.29	1.44	±0.16
27	*p*-cresol					2.25	±0.01
28	phenol					3.81	±0.01
29	*p*-xylene			3.48	±0.12	3.40	±0.13
30	toluene	10.87	±0.14	10.68	±0.30	10.15	±0.31
31	*trans*-isoeugenol	2.72	±0.31				
32	vanillin	1.34	±0.12	1.21	±0.16		

Table 4 shows that 1,3-xylene achieved the highest % peak area (11.02%) among all compounds at 450 °C, and decreased by increasing temperature, stabilizing at approximately 6% peak area. Toluene did not vary with the temperature, presenting an average of 10.57% peak area. The 2-acetyloxyacetic acid was not detected at 450 °C but presented a 7.12% peak area at 650 °C. The ethyl 2-acetyloxyacetate presented a 6.94% peak area at 450 °C, but it was not detected at the highest temperature.

For the HC group, benzene yield did not vary with an average of 3.73% peak area. The alkylated benzenes 1-ethyl-2-methylbenzene and 1,3,5-trimethylbenzene decreased by increasing temperature, presenting maximum values of approximately 4% peak area. For the AD group, although it was not identified at 450 °C furfural presented a 4.27% peak area at the highest temperature. Furan-3-carbaldehyde had a maximum value of 4.08% peak area at the lowest temperature but was not detected at 650 °C. The 2(5*H*)-furanone, in turn, increased its % peak area values by increasing temperature, from 2% at 450 °C to ∼4% at 650 °C. The 2-methoxyphenol, in the PH group, and 1,2-cyclopentanedione, in the CE group presented the maximum values of 4.11% and 4.89% peak area, respectively, at 550 °C.

Comparing the CG-ZeHi and CG-ZeHe experiments, acetic acid, the main compound in CG-ZeHe, was only detected at the lowest temperature in CG-ZeHi, with a 2.88% peak area. Toluene increased by an average of 66% in the CG-ZeHi experiments. The o-xylene, an important compound in CG-ZeHe, was not detected in CG-ZeHi. However, 1,3-xylene was detected in CG-Ze-Hi. The acetol was not detected in CG-ZeHi, but it was an important compound in CG-ZeHe.

Gamliel et al.^75^ studied catalytic fast pyrolysis of *Miscanthus* × *giganteus* by varying some pyrolysis conditions to improve the deoxygenation capacity of liquid products and reduce the production of solids. The authors used ZSM-5 catalysts loaded with metal (Ni), high pressure (2–450 psi), and reactive hydrogen atmosphere. The results showed that the presence of hydrogen did not significantly affect the distribution of products at atmospheric pressure. The liquid yield remained essentially constant for all catalysts at all pressure range tested. However, at high pressures and for Ni catalysts, the permanent gases increased, especially CH_4_, and the solids strongly decreased. Furthermore, the selectivity for monocyclic HCAr increased in parallel with alkanes yield.

Wang et al.^76^ carried out fast pyrolysis experiments of lignocellulosic biomass in a fixed bed reactor in hydrogen and nitrogen atmospheres with/without HZSM-5 zeolite to investigate the influence of pyrolytic agents and updating of volatiles on a zeolite catalyst. The reaction temperature was 450 °C under atmospheric pressure. They achieved higher yields of nonaqueous liquids and permanent gases in the hydrogen atmosphere than nitrogen atmosphere. Catalytic pyrolysis using HZSM-5 zeolite in a hydrogen atmosphere increased the production of polycyclic HCAr and suppressed the production of monocyclic HCAr compared to similar experiments carried out in a nitrogen atmosphere. In general, the hydrogen atmosphere increased the energy content of pyrolytic liquids during catalytic and noncatalytic pyrolysis, which is highly beneficial to applying bio-oil as liquid fuel.

Santana, Menezes, and Ataíde^26^ investigated the fast hydropyrolysis of two types of industrial Kraft lignin at 450, 550, and 650 °C, under 100 psi pressure, and using a Py-GC/MS system. They evaluated the effect of operating temperature and the addition of two acid catalysts (ZSM-5 and HY-340) on the HC production. The fast hydropyrolysis of pure lignin 1 and 2 showed a high formation of PH and an absence of HC. All catalytic fast hydropyrolysis experiments using ZSM-5 promoted a significant formation of HCAr, reaching maximum values of 98 and 99% peak area for lignin 1 and 2, respectively, and *o*-xylene and toluene were the main compounds. The addition of HY-340 led to a high formation of aliphatic HC, with 93 and 92% peak area for lignin 1 and 2, respectively, and hexadecane and tridecane were the main compounds. The other variables studied only slightly modified the selectivity of the products.

Chandler and Resende^77^ compared catalytic fast pyrolysis and catalytic fast hydropyrolysis of giant sugar cane using HZSM-5 catalyst to produce liquid fuels for transportation and understand the effect of hydrogen on the yield and composition of these fuels. The experiments were carried out in a fluidized bed reactor at 400 °C and a pressure of 35 bar since, according to the authors, low hydrogen pressures have little or no effect compared to an inert atmosphere. The hydrogen addition increased the gaseous olefins (C2 – C3) by 540% and the liquid hydrocarbons by 44% while decreasing the coke by 29%. They further estimated that catalytic fast hydropyrolysis could produce 8.9 gal/ton of liquid hydrocarbons (mainly monocyclic HCAr) compared to 6.0 gal/ton in catalytic fast pyrolysis.

He et al.^78^ compared fast pyrolysis, fast hydropyrolysis, and catalytic fast hydropyrolysis poplar sawdust and rice husk to obtain HC. The experiments were carried out at 500 °C, with a Py-GC/MS system and Rh/ZrO_2_ as a catalyst. The yield of volatile products for fast hydropyrolysis of poplar sawdust (75.52% by weight) was close to the fast pyrolysis process (75.62% by weight), but lower than the catalytic fast hydropyrolysis process (85.49% by weight). For rice husk, the volatile products were lower in all pyrolysis processes. The fast hydropyrolysis has a significant hydrodeoxygenation effect compared to fast pyrolysis. The catalytic fast hydropyrolysis showed an additional deoxygenation effect with the highest HC selectivity of 49.14% (33.28% HCAr and 15.86% nonaromatic HC), which presents a better potential for application in biofuels production with low oxygen content.

**Figure 7:**
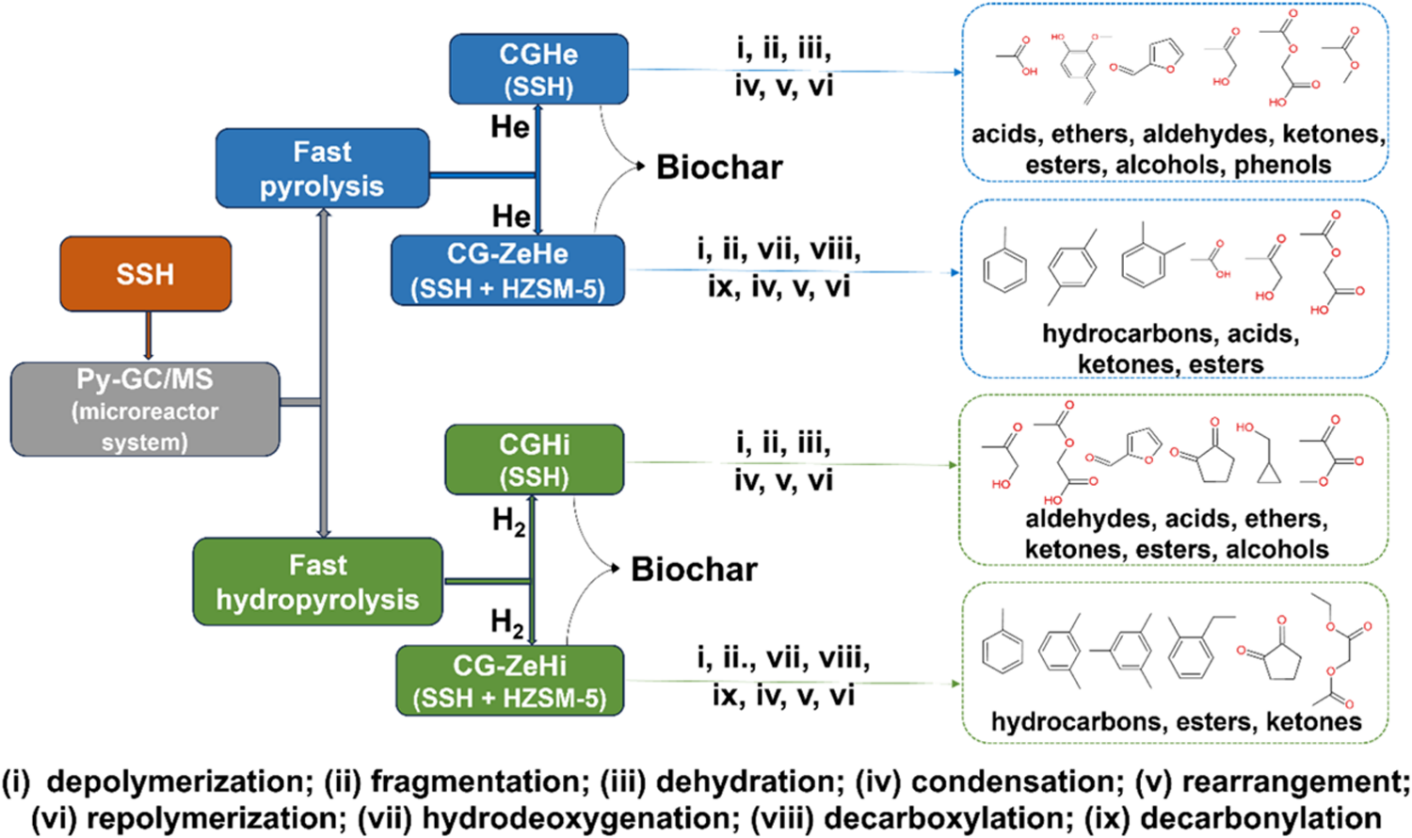
Schematic view of the pyrolysis of SSH and its products.

Figure 7 shows a schematic view of the four pyrolytic conditions to convert SSH, with their main reactions and respective products. The fast pyrolysis and fast hydropyrolysis, without catalysts, provided similar and almost exclusively oxygenated compounds. However, the presence of HZSM-5 zeolite favored deoxygenation reactions of the volatile products, and the catalytic fast hydropyrolysis condition presented the highest deoxygenation.

Biomass pyrolysis aims to make better use of this material converting it into energy and products, which are increasingly demanded by society in its technological and economic development. In addition, it also aims to solve improper waste disposal. In this study, the pyrolysis of SSH under different operating conditions provided organic volatile products that could compose the complex mixture of bio-oil compounds. There is a high demand for HCAr products due to their high-value-added and applicability in transport fuels and the chemical industry, which can be the main target for products to be extracted from biomass. However, oxygenated products also have their value and demand for application in various areas, despite adding undesirable characteristics to bio-oil. Finally, SSH can be used in thermal conversion processes to obtain energy and organic-based products.

## 4 Conclusions

Fast pyrolysis in an inert atmosphere (CGHe) showed a higher fraction of volatile compounds at the highest reaction temperature (650 °C). The main organic groups in the volatile products were ET and AR but only traces of HC. The selectivity distribution for the individual organic compounds showed that acetic acid and 1-hydroxy-2-propanone (acetol) were the main products.

Fast pyrolysis in a hydrogen atmosphere (CGHi) showed a higher % peak area (57%) of volatile products than CGHe at 450 °C. However, at other temperatures, the % peak areas were similar. The organic groups in the volatiles were on average similar to CGHe pyrolysis, except for the decrease in CA (64%) and increase in EL (86%). Acetol, 2-acetyloxyacetic acid, furfural, and methyl pyruvate were the main products.

Catalytic fast pyrolysis in an inert atmosphere (CG-ZeHe) favored the production of volatile products compared to CGHe, especially at 550 °C. The catalyst decreased the % peak area for almost all organic groups present in volatile products, except for AR. In addition, it produced HC with an average of 25% peak area, which would improve the properties of a possible bio-oil production. Among the organic compounds identified, acetic acid, acetol, toluene, *o*-xylene, and *p*-xylene were the main products.

Catalytic fast pyrolysis in a hydrogen atmosphere (CG-ZeHi) presented a maximum % peak area at 650 °C. Compared to CG-ZeHe experiments, it achieved a higher % peak area at 450 °C, but lower values at 550 °C. The main organic groups were AR and HC. Compared to CG-ZeHe, the HC and EL groups showed higher % peak area of 60 and 77% respectively. The CA group reduced by 78% peak area. Toluene and 1,3-xylene were the main products.

However, the zeolite catalyst showed a significant effect on the organic compounds in the volatile products and distribution of selectivity, especially for the formation of HC. This compound may help to promote deoxygenation reactions of pyrolytic volatiles, improving the volatile products. The reaction temperature and gaseous atmosphere played secondary roles. The higher temperature (650 °C) the higher amounts of volatile products. The hydrogen atmosphere presented similar results to the helium atmosphere. These results indicate that this lignocellulosic biomass residue (SSH) can be used as a source of energy and chemical products highly demanded by society. This biomass represents a cleaner and more sustainable alternative to fossil resources. It is produced on a large scale in Brazil, and its thermochemical properties are still little investigated.

## Supporting Information Available

Proximate and compositional analysis of biomasses (PDF)

## Abbreviations

SSH: sunflower seed husks
HZSM-5: proton exchanged Zeolite Socony Mobil-5
CGHe: analytical fast pyrolysis of pure SSH in a helium atmosphere
CGHi: analytical fast pyrolysis of pure SSH in a hydrogen atmosphere
CG-ZeHe: analytical fast pyrolysis of SSH with ex situ HZSM-5 zeolite in a helium atmosphere
CG-ZeHi: analytical fast pyrolysis of SSH with ex situ HZSM-5 zeolite in a hydrogen atmosphere
Py-GC/MS: micropyrolysis coupled to gas chromatography–mass spectrometry
NH3-TPD: temperature-programmed ammonia desorption
AL: alcohol
KE: ketones
CA: carboxylic acids
HC: hydrocarbons
ET: ethers
AR: aromatics
EL: esters/lactones
AD: aldehydes
NI: nitrogenous
PH: phenols
OT: others
HCAr: aromatic hydrocarbons
B/C ratio: biomass to catalyst ratio
