# Supplementary material for: Analytical Pyrolysis of Sunflower Seed Husks: Influence of Hydrogen or Helium Atmosphere and Ex Situ Zeolite (HZSM-5) Catalysis on Vapor Generation
Source: ACS Omega. 2025 Jan 24;10(4):3851–67. doi: 10.1021/acsomega.4c09416 (PMC11800008; doi:10.1021/acsomega.4c09416)
Supplement: Supplementary file 1 — ao4c09416_si_001.pdf [file ao4c09416_si_001.pdf]

Supporting information

Analytical pyrolysis of sunflower seed husks: influence of hydrogen or helium atmosphere and ex-situ zeolite (HZSM-5) catalysis on vapor generation

[1‡]Arthur T. F. A. Araújo; [2‡]Anderson L. de Menezes; [3‡]Cássia R. Cardoso; [1‡]Daniel A. Cerqueira*

[1] Department of Chemistry, Federal University of Triângulo Mineiro, Uberaba, Minas Gerais, Brazil 38064-200

[2] Faculty of Chemical Engineering, Federal University of Uberlândia, Campus Santa Mônica, Uberlândia, Minas Gerais, Brazil 38408-100

[3] Department of Food Engineering, Federal University of Triângulo Mineiro, Uberaba, Minas Gerais, Brazil 38064-200

Table S1. *Proximate and compositional analysis of biomasses.*

Biomass	Proximate analysis (%)			Chemical composition (%)			References
	VM*	ASH	FC*	E*	L*	H*	
SSH (used in this study)	78.15	7.35	14.49	19.09	10.86	68.93	[1,2]
SSH	84.70	3.60	11.70	13.80	31.40	62.50	[3]
SSH	79.80	2.10	18.10	12.00	20.00	57.00	[4]
SSH	76.20	4.00	19.8	2.70	17.00	83.00	[5]
Coffee husk	77.09	3.55	19.36	20.53	27.14	47.29	[6]
Pecan nutshell	67.93	2.47	29.59	9.05	48.37	42.58	[7]
Rice husk	58.22	24.71	8.48	11.51	41.08	47.41	[7]

* VM (Volatile matter); FC (Fixed carbon); E (Extractives); L (Total lignin); H (Holocellulose)

References

(1) Tibola, F. L. PIRÓLISE DAS CASCAS DE GRÃOS DE GIRASSOL – UM ESTUDO SOBRE A CINÉTICA DE REAÇÃO E SOBRE OS PRODUTOS GERADOS, Universidade Federal do Triângulo Mineiro, Dissertação (Mestrado em Química). Uberaba, MG., 2019. https://doi.org/10.1017/CBO9781107415324.004.

(2) Tibola, F. L.; Oliveira, T. J. P.; Ataíde, C. H.; Cerqueira, D. A.; Sousa, N. G.; Cardoso, C. R. Temperature - Programmed Pyrolysis of Sunflower Seed Husks : Application of Reaction Models for the Kinetic and Thermodynamic Calculation. *Biomass Convers. Biorefinery* **2022**. https://doi.org/10.1007/s13399-021-02297-w.

(3) Haykiri-Acma, H.; Yaman, S. Effect of Biomass on Burnouts of Turkish Lignites during Co-Firing. *Energy Convers. Manag.* **2009**, *50* (9), 2422–2427. https://doi.org/10.1016/j.enconman.2009.05.026.

(4) Casoni, A. I.; Gutierrez, V. S.; Volpe, M. A. Conversion of Sunflower Seed Hulls , Waste from Edible Oil Production , into Valuable Products. *J. Environ. Chem. Eng.* **2019**, *7* (1), 102893. https://doi.org/10.1016/j.jece.2019.102893.

(5) Demirbas, A. Effect of Temperature on Pyrolysis Products from Four Nut Shells. *J. Anal. Appl. Pyrolysis* **2006**, *76* (1–2), 285–289. https://doi.org/10.1016/j.jaap.2005.12.012.

(6) Setter, C.; Silva, F. T. M.; Assis, M. R.; Ataíde, C. H.; Trugilho, P. F.; Oliveira, T. J. P. Slow Pyrolysis of Coffee Husk Briquettes: Characterization of the Solid and Liquid Fractions. *Fuel* **2020**, *261* (October 2019). https://doi.org/10.1016/j.fuel.2019.116420.

(7) Davi, G.; Alves, F.; Constantino, J.; Di, M.; Arias, S.; Geraldo, J.; Pacheco, A.; Marangoni, C.; Antonio, R.; Machado, F.; Bolzan, A. Prospecting Pecan Nutshell Pyrolysis as a Source of Bioenergy and Bio-Based Chemicals Using Multicomponent Kinetic Modeling, Thermodynamic Parameters Estimation, and Py-GC / MS Analysis. *Renew. Sustain. Energy Rev.* **2022**, *153*, 111753. https://doi.org/10.1016/j.rser.2021.111753.
